# The Kinesio Taping Method for Myofascial Pain Control

**DOI:** 10.1155/2015/950519

**Published:** 2015-06-21

**Authors:** Wei-Ting Wu, Chang-Zern Hong, Li-Wei Chou

**Affiliations:** ^1^Department of Physical Medicine and Rehabilitation, China Medical University Hospital, Taichung 40447, Taiwan; ^2^Department of Physical Therapy, Hungkuang University, Taichung 43302, Taiwan; ^3^School of Chinese Medicine, College of Chinese Medicine, China Medical University, Taichung 40402, Taiwan; ^4^Research Center for Chinese Medicine & Acupuncture, China Medical University, Taichung 40402, Taiwan

## Abstract

Many people continue suffering from myofascial pain syndrome (MPS) defined as a regional pain syndrome characterized by muscle pain caused by myofascial trigger points (MTrPs) clinically. Muscle spasm and block of blood circulation can be noticed in the taut bands. In the MTrP region, nociceptors can be sensitized by the peripheral inflammatory factors and contracture of fascia can also be induced. Traditional treatments of MPS include stretching therapy, thermal treatment, electrical stimulation, massage, manipulation, trigger points injection, acupuncture, and medicine. However, the pain syndrome may not be relieved even under multiple therapies. Recently, the Kinesio Taping (KT) method is popularly used in sports injuries, postoperative complications, and various pain problems, but little research is focused on MPS with KT method. In this paper, we review the research studies on the application to KT in treating MPS and other related issues. It appears that the KT application can elevate the subcutaneous space and then increase the blood circulation and lymph fluid drainage to reduce the chemical factors around the MTrP region. Therefore, it is suggested that KT method can be used as a regular treatment or added to the previous treatment for myofascial pain.

## 1. Introduction

### 1.1. Myofascial Pain Syndrome (MPS)

Myofascial pain syndrome, defined as muscle pain due to myofascial trigger points (MTrPs) [1], has been considered to be related to poor postures, neuromusculoskeletal disorders, or systemic diseases [2]. Besides, chronic repetitive minor muscle strain, bursitis, enthesopathy, arthritis, or disc lesion can also induce MPS [2]. Clinically, patients with myofascial pain complain about local pain in the muscle, often with referred pain. If the associated pathologic reasons are not well treated, the pain often recurs later [2]. When performing physical examination, the MTrP in a taut band of skeletal muscle can be palpated and local twitch response can be elicited by snapping of the MTrP [3]. Other symptoms of myofascial pain include range of motion (ROM) limitation, sooner exhausting, and referred spasm.

### 1.2. Myofascial Trigger Point (MTrP)

For the diagnosis of MTrP, “spot tenderness,” “taut band,” and “pain recognition” are suggested as the three basic criteria, and “referred pain” and “local twitch responses” are the “signs” for it [4]. In patients suffering from MPS, both latent and active MTrPs may be noted, with characters of spontaneous pain sensation or pain in response to the muscle movement in active MTrPs and tender without spontaneous pain sensation in latent MTrPs. Patient with MPS begins with one active MTrP, called primary MTrP, in the affected muscle due to reasons mentioned above. When under inappropriate treatment, expanding of pain region and additional active MTrPs, called secondary or satellite MTrPs, will develop [1].

## 2. Hypothetical Mechanism of Myofascial Pain Syndrome

### 2.1. Etiology of Myofascial Trigger Point

Acute muscle overload can activate MTrPs. If the lesion is not well controlled, progressive scar tissue will be formed and become a chronic lesion. It may be the major cause of degeneration and activation of MTrP in later life [5].

### 2.2. Integrated Hypothesis of MTrP

In the hypothesis of MTrP as energy crisis postulated by Simons and Travell [6], they have considered “excessive acetylcholine releasing,” “sarcomere shortening,” and “increasing of sensitizing substances” as the three essential characters for the formation of MTrP [7]. An MTrP is composed of multiple contraction knots with sarcomeres overcontracture and increased diameter of that muscle [8, 9]. An MTrP contains multiple sensitive loci suspected as nociceptors and active loci in neuromuscular junctions activated with excessive acetylcholine leakage even under relaxation in the nonendplate zone [10]. In this condition, the sarcomeres in the endplate zone will contract continually and form the contraction knot in the endplate zone and the taut band in the whole muscle fiber, the pain threshold of nociceptors will be decreased, and the symptoms become severe. When energy crisis occurs, ischemic tissues lack adenosine triphosphate to promote calcium pump in the sarcoplasmic reticulum. It will make the muscle contract continually with regional sarcomere shortening and then blocks the supplements of nutrition and oxygen moreover [7, 11]. Local ischemia and hypoxia can induce secretion of sensitizing substances to cause pain and release abnormal acetylcholine resulting in a vicious cycle [7].

### 2.3. Clinical Researches of MTrP

To investigate the pain, research of MacDonald [12] showed that muscles with active MTrPs have restricted passive ROM. Since the tension of involved muscle fibers has been increased even at rest, stretching the muscle beyond limitation can produce severe pain. Painful contraction can be also noted when performing test of fixed resistance [12]. After treating the MTrPs and releasing the taut band, the ROM can be returned to original status.

In surface electromyographic (EMG) studies performed by Headley [13, 14], it was found that muscles with active MTrPs beginning fatigue, exhausting sooner, and recovering later than normal muscles. Another research using surface EMG for endurance test on myofascial pain demonstrated that amplitudes of EMG activity increased and mean power frequency decreased with time. And on the more painful side, accelerated fatigability was noted with shorter duration of endurance comparing to the normal side [15]. In a research with surface EMG, it was shown that the muscle containing active MTrPs was under status of fatigue, and exhausting the energy earlier than the normal one [16]. Besides, after injection of 2% lidocaine solution on trigger points of upper trapezius muscle, significant reduction in pain intensity (*P* < 0.001) and EMG activity (*P* < 0.03) in ipsilateral masseter muscle was noted with confirmation of referred spasm due to myofascial pain [17].

In consideration of image finding, Sikdar et al. apply ultrasound on taut band in the upper trapezius [18]. They found MTrPs as focal, hypoechoic regions and reduced vibration amplitude under vibration sonoelastography. It indicated local changes in tissue echogenicity and appeared with elliptic nodules of size about 0.16 ± 0.11 cm^2^. Chen et al. [19], through MRI study, noted that stiffness of the taut band in patients with myofascial pain was about 9.0 kPa and 50% greater than surrounding tissue.

To confirm the theory of energy crisis, Shah and his colleagues used micropipettes to determine the pH value and the electrolyte concentration in both active and latent trigger points and also in control points [20, 21]. They found significant higher concentration of inflammatory mediators (like bradykinin, substance P, tumor necrosis factor-alpha, interleukin-1 beta, serotonin, and norepinephrine) and lower pH value in the active or latent MTrPs regions than those in the normal points. These inflammatory mediators can induce peripheral sensitization of nociceptors in muscle or central sensitization in central nervous system. When the message was transferred to spinal cord through nociceptors, it can induce neural circuits of MTrP in central nervous system and can form latent MTrPs in muscle [2, 21, 22]. When increasing the stimulation to this neural circuit due to acute or chronic injury, latent MTrPs can be activated into painful active MTrPs.

Researches conducted by Mense [23, 24] for central sensitization reported that persistent stimulation of sensory afference from muscles would lead to neuroplastic changes in the posterior horn of the spinal cord and allodynia often associated with active MTrPs. Releasing of substance P, glutamate, and calcitonin gene-related peptide from the primary afferent fibers can sensitize the nociceptors either at receptive or spinal ends. These neuropeptides will also enter into other synaptic associations with other posterior horn neurons with consequence of hyperalgesia. Besides, nociceptors near the site of pathology can transmit messages to neural connections of associated MTrPs and then induced the latent MTrPs to active MTrPs.

## 3. Treatment of Myofascial Trigger Point

Due to multiple factors of MPS, single management or therapy may not overcome the problem effectively. Treatments of MTrP include manual therapies [1], physical therapy modalities [25], needling therapy (including MTrP injection [26], dry needling [27–29], acupuncture [30–32], percutaneous soft tissue release [33], and subcutaneous needling [34]), or oral medicine. Eliminating any perpetuating factors and introducing adequate education and home programs to patients are also important [1, 35].

The earliest effective therapy suggested for treatment is spraying ethyl chloride on skin combined with stretch [1]. Travell suggested applying two or three sweeps of spray before or concurrently while gently stretching the muscle to its full length [36]. But due to the side effects, such as respiratory tract injury, freezing, and environmental destruction, the spray was displaced with ice rubbing.

In exercise therapy, Lewit and Simons [37] introduced postisometric relaxation (PIR) exercise as a treatment. Patients perform isometric contraction on those muscles with 10–25% of full strength. Then they make the patient relax the muscle three to ten seconds later, following mild stretch of the same muscle by clinician, and relax again. The circle shall be performed for several times. When combining PIR exercise with reflex augmentation of relaxation including respiration and eye movement, the effectiveness will be highly enhanced [38].

Cyriax [39] developed a deep fraction massage requiring that the finger runs across the long axis of muscle fibers or taut bands at level of MTrPs, and it is specific for those located at middle of muscle belly. Rolfing method introduced focuses on viscoelasticity of the fascia [40]. By this manual treatment, firm type of colloid fascia due to mechanical perturbation can be transduced to a more liquid form. The fascia contains abundant innervation with mechanoreceptors. Fascia releasing technique with stimulation of Golgi receptors can lead to changes in the underlying tension of the skeletal muscle. At least, by increasing local proprioception, status of dysfunction will be reduced.

Recently, few studies researched the therapeutic effect of Kinesio Taping (KT) method as a new therapy of MPS and with hope of self-application for this condition.

## 4. Kinesio Taping (KT) Method for Myofascial Pain Syndrome

### 4.1. Background of KT Method

The concept of KT originated from the tradition athletic taping. Traditional athletic taping, with thick, sticky, and firm material, has been developed since 1882. By restricting the ROM through immobilizing and stabilizing joints or muscle, the tape can prevent secondary injury effectively, reduce edema and pain, and completely immobilize the treatment area.

KT method was developed from 1973 to 1979 by Dr. Kenzo Kase, in an intension to provide support for musculoskeletal structure without overimmobilization and the side effect from it. Kase commercially introduced the tape for KT in 1982, with elastic, cohesive, lightweight, and ventilation characters. The original purpose was for edema control, soft tissue support, joint protection, and relieving heat produced from active inflammation. Advanced purpose was continuing the effect of manual therapy from clinic to home care and activity of daily living. After the application of KT for Japanese athletics at the Seoul Olympics in 1988, this method achieved worldwide concerns and then was introduced to the United States to become popular rapidly.

### 4.2. Characters of KT

The name Kinesio for this woven-cotton and elastic tape is originated from the word “kinesiology,” since the tape applies over and around muscles for movement control and functional goals. This tape, which is with elastic core wrapped within cotton and capable of stretching up to 140–150%, applies heat-sensitive acrylic adhesive to avoid risk of latex allergy especially used in children. The tape without medicinal properties is water-resistant and can remain on the skin for 3 to 5 days. Special design with waved structure can alternate the inputs of proprioception and somatosense. This elastic tape can be performed or cut into special pattern for any alignment of the human body easily.

## 5. KT Method for Pain from MTrPs: Clinical Trials and Basic Research

### 5.1. Clinical Researches of Fascia

Fascia can be separated into superficial and deep layers under traditional concepts. The superficial layer, composed of loose connective and fat tissue, locates between skin and muscle layer. Blood vessels, lymph nodes, nerves, fluid, and gel-like matrix are intersecting in this layer. The superficial layer must be soft enough, and cells like mast or white blood cells can cross over for reason of defense, nutrition, support, hot, and metabolite exhausting. The deep layer, with functions of protection and stabilization, is composed of firm and intimate collagen fiber to separate different muscles, nerves, and organs.

Multiple myofibroblasts locate near the capillary vessels in fascia and are capable of offering enough contractile force [41]. These cells will not be affected by norepinephrine, acetylcholine, or angiotensin, while sustained tension going through the tissue, nitric oxide, histamine, and oxytocin will induce longer duration but lower energy contraction [42]. Lower pH level in matrix tends to increase contractility of myofibroblasts and then induces a general stiffness of the fascia [43].

Regarding the new concept of the structure of fascia in recent years, Guimberteau et al. [44] developed the multimicrovacuolar collagen dynamic absorbing system (MCDAS). This is quite different from the traditional concepts of fascia with firm, sticky, dehydrated structure obtained from the anatomy sample. Microvascular tube are filled with hydrophilic jelly made of proteoglycoaminoglycans. They are limited but crossing over in hollow fibrils made of collagen and elastin. In order to keep being in balance, structures of microvacuolar can separate, blend, reform, and roll over each other in response to all forces from osmotic pressure, surface tension, weight, and gravity.

For this reason, the structure of fascia in the whole body is continual and allows multiple sliding directions to correlate the construction of skin, nerve, vessels, and muscles. Because capillaries cross over in fascia, circulation will be involved if the fascia becomes retardant, degenerated, and stiff. Since previous researches [20, 21] reported higher concentration of inflammatory mediators and lower pH value in the active or latent MTrPs regions, recirculation through realignment and decompression of the fascia will help relieve the symptoms.

### 5.2. Basic Researches of KT Method

**Figure 1:**
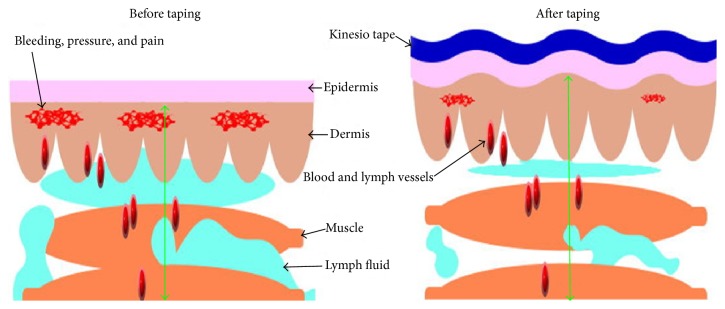
These two pictures showed the mechanism of KT application on soft tissue. Before taping, the lesion site, such as a taut band or bruise, may contain bleeding, pressure, and lymph fluid accumulation and then cause pain. After taping as shown with blue curve, the space-lifting mechanism will help the drainage of fluid. Then the inflammation factors and pressure can be reduced, and the movement of muscle can be improved.

Main purpose of KT method as in Figure 1 is elevating the space under skin and soft tissue, so that the space for movement can be enlarged, the circulation of blood and lymph fluid can be facilitated, and healing rate of tissue can be increased [45]. To confirm this hypothesis, Shim and his colleagues [46] reported a study with rabbits about wrinkles not only compressing the skin, but also elevating the space. They announced positive effect on opening microvalves due to dynamic pressure variation. Since periodic compression and decompression to superficial and deep lymphatics, through expansion and contractile properties of the tape during active movement, the flow and circulation were improved.

To confirm this effect, Kase [47] once researched the influence of taping on blood circulation. The participants were randomly tested through ultrasound under Doppler view for radial, superficial temporal, and dorsalis pedis artery before and after taping. It was found that the flow rate was increased immediately after KT.

Bialoszewski and his colleagues [48] studied 24 patients treated with Ilizarov method for lower limb lengthening and complicated with thigh edema. They were divided into two groups. Both groups received 10 days of standard physiotherapy, and additional application of KT was performed in the experimental group. They reported statistically significant decrease in the circumference of thigh and leg in both groups (*P* = 0.02, *P* = 0.03, resp.), with more significance in the experimental group than the control group with only standard lymphatic massage. However, they did not provide statistical data for intergroup comparison research.

Aguilar-Ferrándiz and his colleagues [49] reported an article of treating patients with chronic venous insufficiency for 4 weeks. Participants were randomly assigned into an experimental group for standardized KT application or a control group for sham KT treatment. Only experimental group showed improvements compared to pretreatment values in swelling (*P* < 0.002), muscle cramps (*P* < 0.001), and pain distribution (*P* < 0.001). Intragroup comparisons for the improvement of pain score between baseline and posttreatment were significantly greater in the experimental group than the sham group. But placebo effect should also be taken into consideration because of mild reducing of pain score in control group.

Kalichman et al. [50] reported a case suffering from meralgia paresthetica with symptoms of numbness, paresthesias, and pain in the anterolateral thigh. After using KT method for 4 weeks, symptoms and quality of life were significantly improved.

### 5.3. Mechanism of KT Method on Lymphatic System

In investigation of lymphatic system, a one-way system below the surface of skin, it depends on negative-pressure pumping to guide the fluid flow from superficial to deep layer. This negative-pressure effect is facilitated by the alternative muscle contraction and relaxation. If interstitial pressure around the lymphatic system is much increasing due to edema, intercellular junction doors will be closed [51]. By muscle contraction, relaxation, and therapies such as massage or compression garment, pressure in each segment can be changed. The lymph and interstitial fluid can recirculate and decrease the swelling and pain sensation [52, 53].

Besides, due to the drainage effect of lymph edema, the circulation of lesions can be improved and then accelerates the healing of the tissue. Ristow et al. [54] once researched the application of Kinesio Tex Tape after surgery for mandibular fracture. They announced statistically significant decreasing the incidence of swelling when applying KT during the first 2 days after operation.

### 5.4. Other Probable Mechanisms of KT Method for Proprioception and Pain

Many articles have reported different conclusions of effects on taping, such as proprioception, placebo effect, warning message, or biomechanics. For example, treatment with additional KT method over exercise for patellofemoral pain syndrome could provide significantly better hamstring flexibility than the control group (*P* < 0.05) [55]. On the other hand, a research of athletic tapes also showed that increasing cutaneous sensory feedback would improve ankle joint position perception (*P* < 0.05) under non-weight bearing condition [56].

Besides, some researchers reported that KT method would improve the ROM of neck [57] and lower trunk [58]. For example, Osterhues [59] researched the effect of taping on those who suffered from acute whiplash injury. The study group showed a significantly greater decrease in pain immediately (*P* < 0.001) and also significant increase in ROM in all directions. Osterhues [59] treated patients of patella bony dislocation with KT based on the concept of stimulation to mechanical receptors of skin from taping and found that the balance and motor control were better than previous status. When performing the kneeling down or eccentric contraction of leg under-weight bearing, visual analogue scale (VAS) was decreased.

Effect of pain control of KT method may associate with gate control theory [60]. A*β*-fiber, afferent fiber from sensory neurons of touch, is bigger in diameter and conduction velocity than those for pain including A*δ*-fiber and C-fiber. By stimulating the afferent receptors with light touch in the skin can activate glial cells in the spinal cord. Then transmission of pain will be inhibited at the spinal cord level from transmitting to the cortex. Until now, no research can confirm that KT can provide effectiveness via this theory but can depend on literatures of other physical modalities used for reduction of pain [61, 62]. Other issues such as balance of agonist and antagonist, tendon and ligament protection, decreasing muscle activity of protective contracture, and sensing of movement still require further studies.

### 5.5. Clinical Taping for Facilitation and Inhibition

In researching of facilitation for muscle, Rood facilitation including fast brushing, light moving touch, and icing on fascia is reported as a useful method [63]. On the other hand, the inhibitory pattern is correlated with the Golgi tendon organ (GTO) located at muscle-tendon junction in the insertion end. These methods include lightly compressing the joints, compressing the muscle-tendon junctions, and keeping tendon in stretched condition [64]. GTO, sensitive to changes in muscle tension, connects to muscle fibers and communicates the muscle spindle status. When in action, GTO inhibits its own muscle and excites the antagonist [65].

The input of stimulation for facilitation and inhibition mentioned above can be achieved through taping. By changing proprioception, biofeedback from correct movement pattern, and training or rehabilitation in high intensity, the target muscle groups and coordination can be improved. Then let the patient perform similar suitable movement after removing the tape.

Kaya et al. [66] applied KT combined with home exercise program on patients who suffered from shoulder pain due to impingement syndrome. The tapes were applied with “insertion to origin technique” over the supraspinatus, teres minor, and deltoid muscles. Significant improvement in pain and disability was noted in the taping group one to two weeks later. In Şimşek's research [67], for outcomes in subacromial impingement syndrome with KT in addition to exercise therapy comparing to the sham taping, pain during movement in the therapeutic group was significantly lower at the 5th day (*P* < 0.01) in intergroup comparisons. And night pain, pain with movement, shoulder external rotation muscle strength, and pain-free shoulder abduction ROM in the therapeutic group at the 12th day were significantly improved (*P* < 0.05).

### 5.6. Probable Mechanism of KT Method for MTrPs

**Figure 2:**
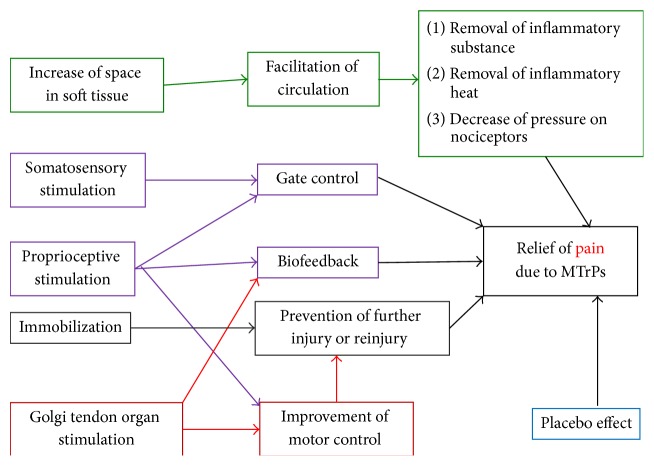
The possible mechanism of Kinesio Taping for the relief of myofascial pain.

The concepts mentioned above can be compatible with the hypothesis proposed by Kase about the space, movement, and cooling effect of taping. Increasing the space of fascia to improve the circulation can remove the heat produced from inflammation. Pain sensation can be diminished due to reduction of the pressure on nociceptors. The theory is similar to that of treatment for MPS [2, 68, 69]; therefore we hypothesize that KT method can block the vicious circle of energy crisis.  Figure 2 showed the possible KT mechanism for the relief of myofascial pain.

### 5.7. Researches of KT Method for Myofascial Pain

Wang and her colleagues [70] investigated the effect of KT method for the relief of MPS. Taping with “insertion to origin technique” was performed on the upper trapezius muscle and statistically significant pain relief was found immediately after the treatment. They considered the effects due to taut band stretching and stimulation of skin receptors. No improvement was reported by the patients in the control group. The improvement of VAS in the experimental group remained statistically significant 24 hours later.

García-Muro et al. [71] reported a patient with shoulder pain of myofascial origin under intervention of KT method. They found that this patient had significant improvement in the VAS, algometry, functional tests, and active ROM, resolving the problem in the following days. Therefore, they announced that KT methods were appropriate for treating MTrPs.

## 6. Clinical Application of KT Method: Prescription and Contraindication

KT method combined with other therapeutic interventions may reduce pain, regulate (facilitate or inhibit) muscle function, provide proprioceptive feedback, and stabilize joint. Before prescribing KT, the practitioner shall understand the past history, posture at daily living, type of work or exercise, biomechanics and duration of injury, and previous treatments and the effects. Detailed physical examination includes inspection, palpation, active and passive ROM, resistant test, and identification of the MTrPs and taut bands. It is also important to consider the “upper and lower crossed syndrome” announced by Janda [72], such as tightness of upper trapezius and pectoralis combined with weakness of rhomboid and anterior serratus. After history taking, examination, and locating the MTrPs, manual therapy or needling (dry needling or injection to MTrPs) can be performed as necessary. For relieving the swelling and tenderness after injection, taping can be used following the guidance for the desired direction of drainage.

The basic principle of prescription of KT for myofascial pain focuses on the patterns of facilitation and inhibition. When the tape applies on the muscle from its origin to the insertion site, it can provide the effect of facilitation to the muscle contraction [47]. On the other hand, when taping from insertion to origin, inhibition and relaxation of muscle spasm will be the effect [47] which is most useful for myofascial pain and muscle spasm.

The contraindication of KT method includes taping on sites of acute infection, open wounds, deep vein thrombosis, malignancy, and severe allergy. Djordjevic et al. [73] suggested checking skin for allergy to the tape by applying a small (1 × 1 cm^2^) patch of tape on the volar side of forearm. The positive finding of this allergy test was redness or other skin changes noted in 15 minutes. If patient suffered from diabetes mellitus, taping shall be carefully used due to possible sensory defect.

## 7. Limitation of Researches on KT Method

Researches for the effect of KT method often show different results. Hsu and his colleagues [74] applied taping on baseball players with shoulder impingement and then measured the EMG activities of the upper and lower trapezius. They found significant increase of the lower trapezius muscle activity in the arm-lowering phase (60–30°, *P* < 0.05) in comparison to the placebo taping. Similar research with increasing bioelectrical activity of vastus medialis muscle was reported by Słupik et al. [75]. Other researchers such as Firth et al. [76] used the Hoffman reflex amplitudes to assess the effect of taping on people with Achilles tendinopathy. They reported that the H reflex remained unchanged in the soleus and gastrocnemius in study group.

Therefore, more research studies are necessary to clarity of the effectiveness of KT method. Many limitations in researches shall be overcome, and it is very difficult in building random or double blind trials. Furthermore, the taping techniques were variable in previous studies. Although all procedures of KT for a similar disorder were performed according to Kase's original concept, different practitioners might perform different technique based on their previous experience and manner and could induce the bias [77].

Another limitation for research is the placebo effect. Some research announced that visual input of different color and the sensation stuck on skin may make the positive expectancy [78] and may make the patients feel confidence, stability, and reassurance [79].

Besides, no suitable machine or image data can confirm the effect of taping at anytime and anywhere. With the functional improvement of ultrasound, certain landmark could be tracked more easily. By using sonography, we can exactly define the depth, certain muscle, and surrounding tissue and note the twitch response during injection. We can not only avoid injury from treatment but also increase the specificity of the target tissue for taping. For example, in cases with epicondylalgia after KT method application, Liu and his colleagues [80] reported improvement of epicondylar muscles sliding in ultrasonic image when wrist was moving. Since the flexibility of tissue correlates with the pathologic status, sonoelastography can be used for identifying the location and may be considered for researching of outcomes after taping.

## 8. Conclusion

In clinical practices, KT method was applied in sports injuries, postoperative complications, various pain problems, and many other conditions. The tape is simple to carry out, economic, and less traumatic. In treatment of patient with MPS who cannot be rehabilitated regularly, some researchers suggested taping through self-application as a new therapy [70]. However, self-application of tape may be difficult in some aspects including the limited knowledge in anatomy or biomechanics, the inadequate knowledge in trigger point examination, the lack of experience of taping method, the requirement of using both hands, and the location of the MTrP (such as rhomboid muscle). Therefore, most people cannot tape by themselves. In order to obtain a better effect, it is also necessary to combine therapeutic exercise, postural changing, and adjustment of daily living. Finally, we considered that KT method could be applied as another choice of MTrP therapy but could need more researches to confirm the effectiveness.
